# A Rare Presentation of Malignant Melanoma in an Adult: Diagnostic and Therapeutic Insights

**DOI:** 10.7759/cureus.75016

**Published:** 2024-12-03

**Authors:** Mithun Chakraverthy Pudota, Sneha Udamala, Guna Sai Vallapuri

**Affiliations:** 1 General Surgery, P.E.S. Institute of Medical Sciences and Research, Kuppam, IND

**Keywords:** chest wall swelling, excision, hydrothorax, malignant melanoma, malignant melanoma metastasis, melanoma, oncosurgery, pyothorax, surgery, tumor

## Abstract

This case presents a rare and aggressive manifestation of malignant melanoma, initially presenting as a chest wall swelling in a young male with a history of trauma and subsequent management for hemothorax and pyothorax. The complexity of this case lies in its atypical presentation and the challenges posed in diagnosis and treatment.

A 30-year-old gentleman presented to the general surgery clinic with a chief complaint of swelling on the right side of his chest, persisting for two months following a traumatic fall, which later resulted in hemothorax and prothorax required drainage and eventually ended up developing a swelling requiring further investigations. Imaging studies, including CT and contrast-enhanced CT, indicated a large heterogeneous mass with lytic destruction of the underlying rib and infiltration into adjacent structures. An incisional biopsy confirmed the diagnosis, which required a wide local excision of the tumor. Intraoperatively, the tumor was found to involve surrounding structures, necessitating extensive resection and reconstruction. After surgical removal, histopathological examination confirmed malignant melanoma with involved margins. Additional studies like immunohistochemistry and PET-CT were required to understand the comprehensive understanding of tumor and its metastasis. Malignant melanoma, though rare, should be considered in the differential diagnosis of atypical chest wall swellings, especially in patients with recent trauma and surgical interventions. This case underscores the aggressive nature and complex management of melanoma, highlighting the necessity for prompt diagnosis and comprehensive treatment strategies to improve patient outcomes.

## Introduction

Malignant melanoma arises from melanocytes, cells originating from neural crest cells and migrating to various tissues including the skin, mucosal surfaces, meninges, and uvea. Although less common than other skin cancers, melanoma is particularly lethal due to its aggressive nature and high metastatic potential. Risk factors include ultraviolet (UV) light exposure, genetic predispositions, and immunocompromised states. It is known for its ability to metastasize rapidly to distant organs and its resistance to conventional therapies, making it one of the deadliest types of skin cancer [1]. The global incidence of melanoma has been steadily increasing over the past few decades, particularly in regions with higher UV radiation exposure [2]. This trend is alarming, as melanoma now represents a significant public health concern, especially in countries with predominantly fair-skinned populations [3]. Several risk factors contribute to the development of malignant melanoma, including genetic predisposition, phenotypic characteristics such as fair skin, and environmental factors like excessive UV radiation exposure [4]. The role of UV radiation in the pathogenesis of melanoma is well-documented, with intermittent, intense exposure, particularly during childhood and adolescence, being a critical risk factor [5]. Additionally, genetic mutations, particularly in the BRAF and NRAS genes, are commonly associated with melanoma and contribute to its pathogenesis [6].

Early detection of melanoma is crucial for improving patient outcomes. The prognosis of melanoma is highly dependent on the stage at diagnosis, with early-stage melanomas having a significantly better prognosis compared to advanced stages [7]. The ABCDE (Asymmetry, Border irregularity, Color variation, Diameter greater than 6mm, and Evolving nature) criteria are commonly used for the clinical evaluation of suspicious pigmented lesions [8]. Despite advancements in the understanding of melanoma biology and the development of targeted therapies and immunotherapies, the management of advanced melanoma remains challenging [9]. This case report aims to provide a detailed account of the clinical presentation, diagnostic challenges, and therapeutic approach in a patient with malignant melanoma, emphasizing the need for a multidisciplinary approach in managing this complex disease [10].

## Case presentation

A 30-year-old gentleman, occupied as a mason from a rural region of South India, presented with a swelling on the right side of his chest. The swelling developed two months after a significant traumatic event, a fall from an 8-foot height at his workplace. Initially managed with over-the-counter medications for cough and breathlessness, he was later diagnosed with right hemothorax and treated with intercostal drainage, followed by pyothorax requiring pigtail catheter drainage. Despite the resolution of the acute issues, the patient noticed a progressively enlarging, painless swelling, raising concerns for a chronic underlying pathology.

The differential diagnosis for a chest wall mass in this context was broad and included both infectious and neoplastic processes. Possible diagnoses ranged from chest wall abscess and empyema necessitans to more ominous conditions such as soft tissue sarcoma, skin tumors, chondrosarcoma, and malignant melanoma. Additionally, conditions like chest wall hematoma, ruptured liver abscess, and liver tumors extending to the abdominal wall were considered.

**Figure 1:**
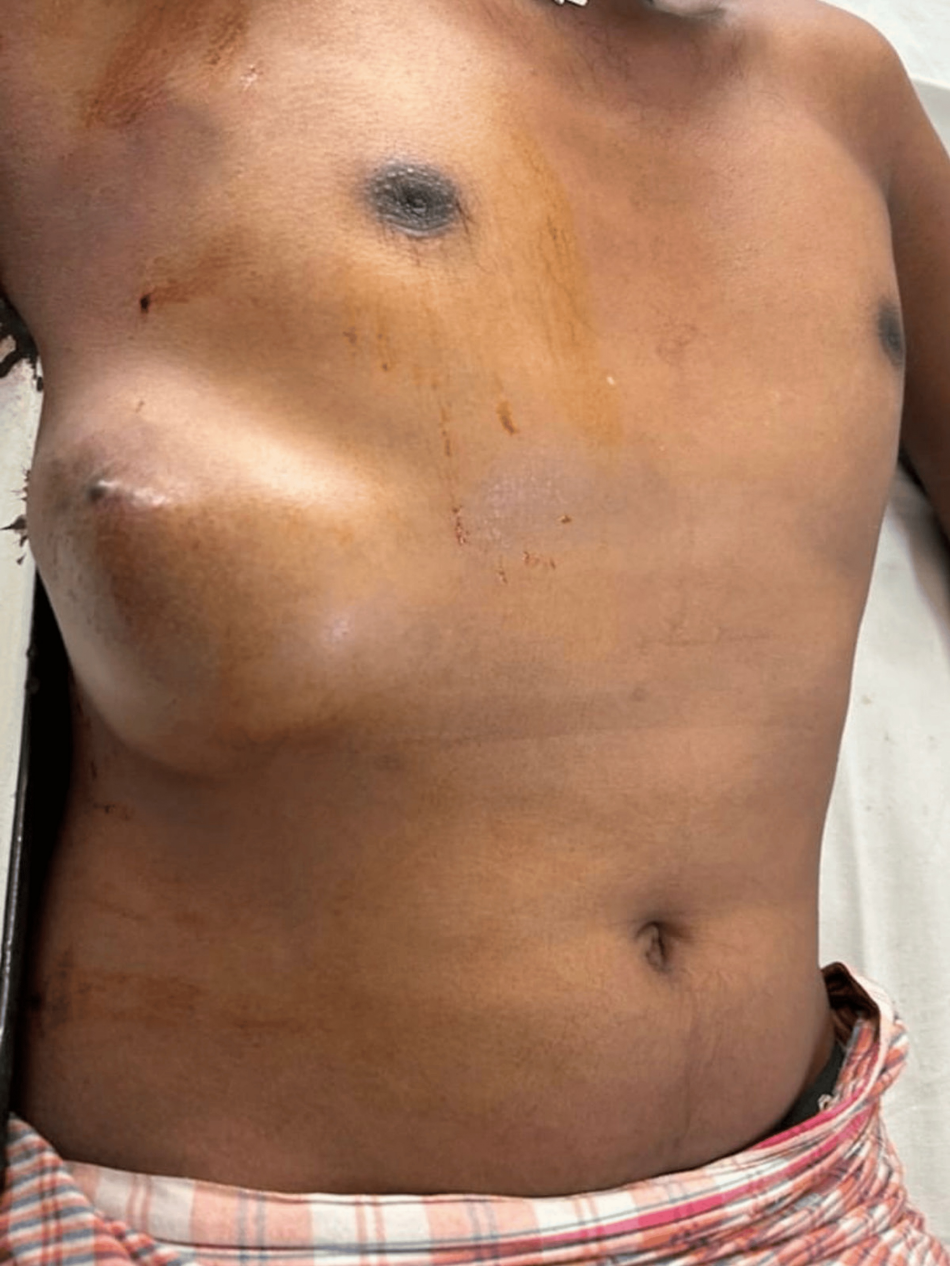
Clinical presentation of the chest wall swelling A solitary, obliquely ovoid swelling measuring 12 x 10 cm is observed on the right side of the chest, extending horizontally from the midclavicular line to the posterior axillary line. A hyperpigmented nodule is present within the lesion.

Physical examination revealed a firm, solitary, immobile, ovoid mass of size 12 x 10 cm with irregular borders extending horizontally between the midclavicular line to the posterior axillary line and vertically ranging from 4 cm below the nipple to 4 cm above the right costal margin. A hyperpigmented nodule was noted to be present over the swelling. The serum studies and blood tests were unremarkable (Figure 1).

Imaging studies

**Figure 2:**
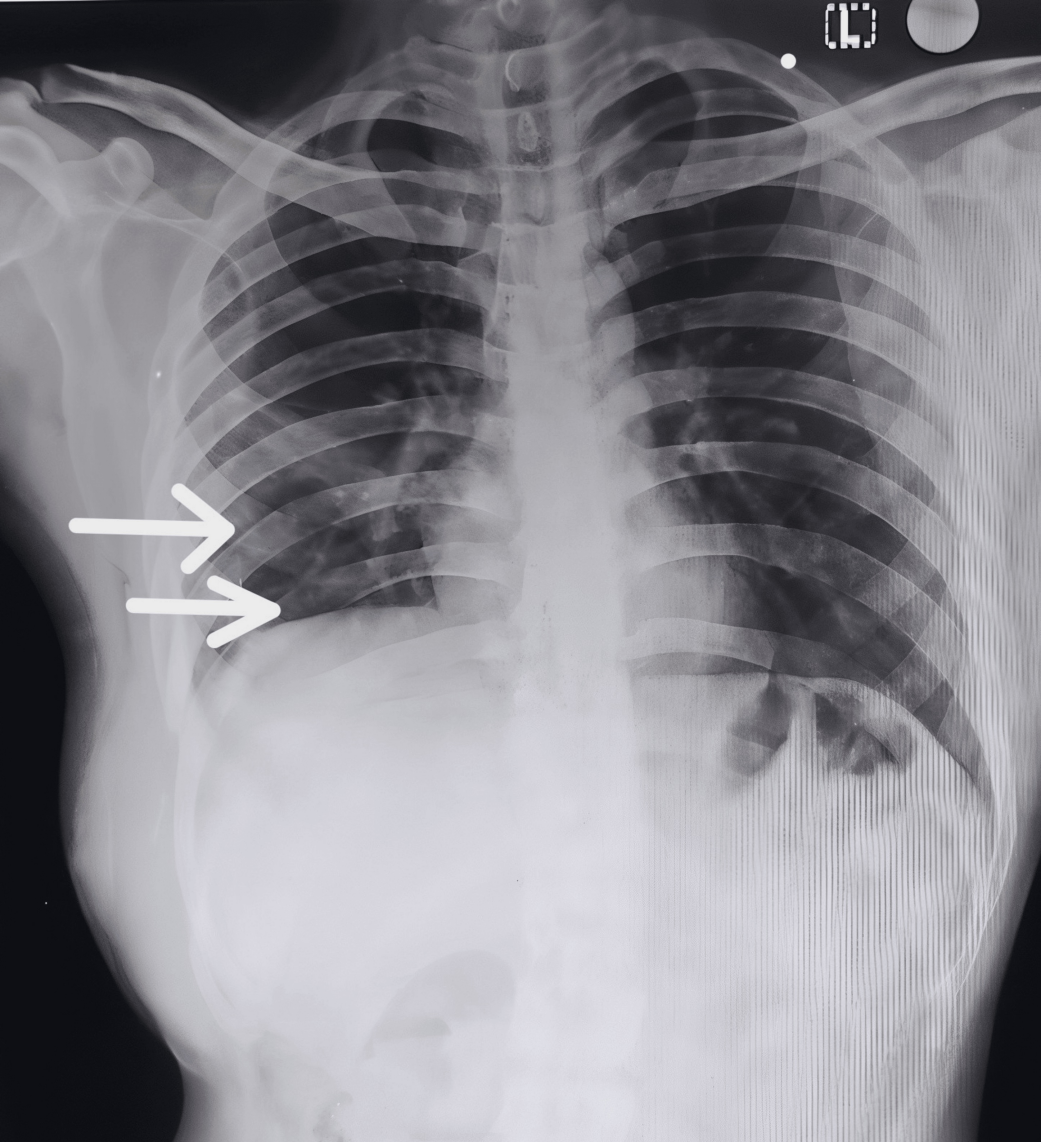
Chest X-ray of a 30-year-old male with suspected local invasion of malignant melanoma The posteroanterior chest X-ray of a 30-year-old male reveals lytic lesions in the eighth and ninth ribs on the right side, indicative of potential local invasion by malignant melanoma (white arrows).

Chest X-ray, CT, and contrast-enhanced CT scans identified a heterogeneous mass with lytic destruction of the underlying eighth and ninth ribs (Figure 2). In addition to this, a close indentation of the liver, suggestive of a neoplastic process, was noted.

The incisional biopsy was crucial, revealing atypical melanocytes and melanin pigment, leading to a diagnosis of malignant melanoma. Given the aggressive nature of the tumor and its extensive local invasion, surgery was scheduled to remove the mass.

Surgical procedure and intraoperative findings

**Figure 3:**
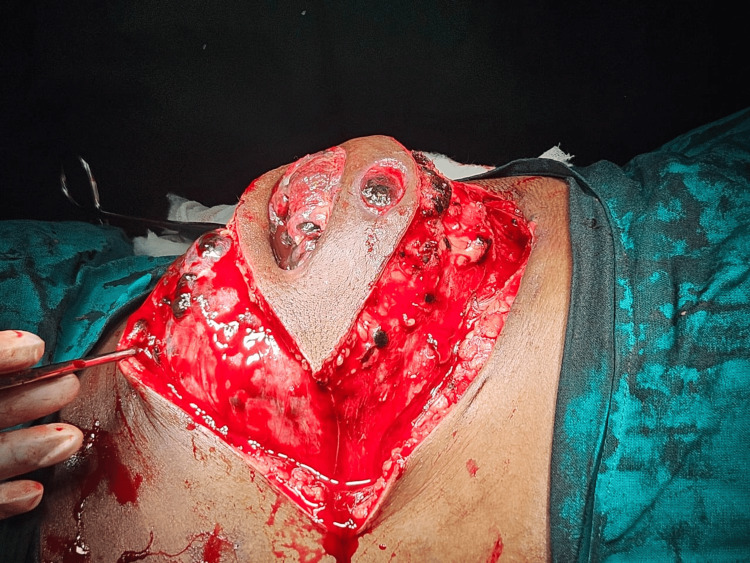
A wide local excision This image shows the surgical excision of the swelling, performed with a 2 cm margin.

**Figure 4:**
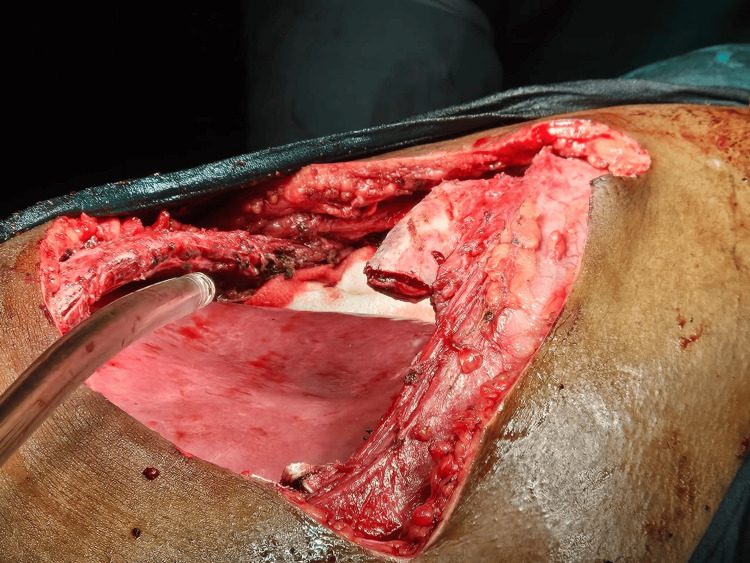
Partial resection of rib This image shows the partial resection of the lytic ninth rib, which was encompassed within the tumor. Additionally, portions of the eighth and tenth ribs were excised as part of the procedure to ensure complete removal of the affected tissue.

**Figure 5:**
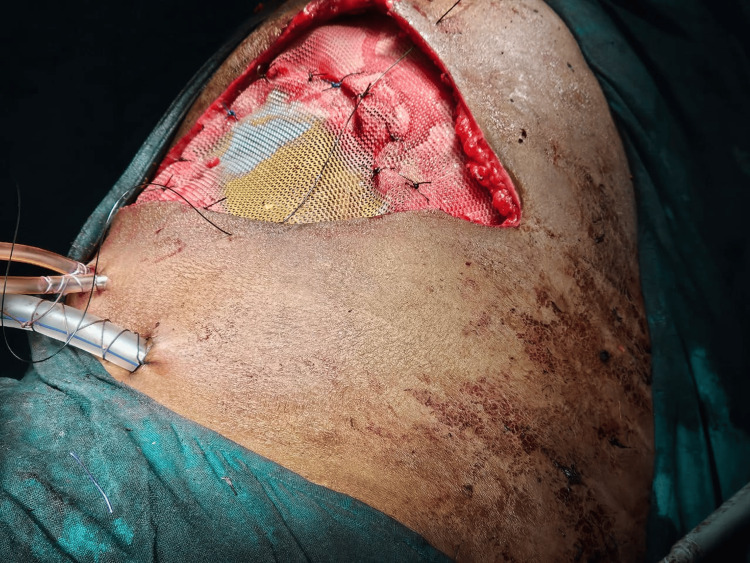
Reconstruction with mesh This image shows the reconstruction of the chest wall using a composite mesh placed below the ribs and a Prolene mesh positioned above the ribs. The surgical site was then securely closed.

A wide local excision with a 2 cm margin was performed. Intraoperative findings were striking, with the tumor infiltrating the diaphragm and peritoneum, and causing lytic destruction of the ninth rib. A comprehensive resection was performed, including the removal of involved ribs and the infiltrated diaphragm, followed by reconstruction with composite and Prolene mesh (Ethicon, Inc., Raritan, USA) (Figures 3-5).

Histopathological examination confirmed the diagnosis of malignant melanoma with Clark level V invasion, indicating deep infiltration into the subcutaneous fat. The presence of microsatellites and involved margins highlighted the aggressive behavior of the tumor. Immunohistochemistry further supported the diagnosis, with HMB 45 positivity in lesional cells and evidence of lung parenchymal infiltration.

Postoperative course

Postoperatively, the patient’s prognosis remains guarded due to the extensive nature of the disease and involved surgical margins. PET-CT revealed metabolically active deposits consistent with metastases in the mediastinum, pelvis, retroperitoneum, pleural bases, and potential involvement of the anorectum. This underscores the need for close follow-up and consideration of adjuvant therapies, including targeted therapy and immunotherapy.

This case underscores the importance of considering malignant melanoma in the differential diagnosis of unusual chest wall swellings, especially in patients with a history of trauma and recent surgical interventions. The aggressive nature of melanoma, coupled with its high metastatic potential, necessitates prompt and comprehensive diagnostic and therapeutic approaches. Early identification and intervention are crucial to improving outcomes in patients with this formidable malignancy.

## Discussion

This case is significant due to its rare presentation of malignant melanoma in a young male with a preceding history of trauma and subsequent hemothorax and pyothorax. Unlike the more common cutaneous presentations [11], this patient’s melanoma manifested as a chest wall mass, highlighting an atypical pathway of disease progression. Comparatively, most reported cases of melanoma arise from pre-existing nevi on the skin [12], with chest wall involvement being exceptionally uncommon. Although cutaneous malignant melanoma presentation can present as lymphadenopathy [13], mass effects causing edema [14] pose a difficulty in diagnosing the condition but the presentation in this young male, who was asymptomatic prior to the trauma. Additionally, this patient did not show any signs of the diagnosis like patches [15] or nodules [16], which made the clinicians not include the diagnosis in differentials. This deviation from the norm emphasizes the importance of considering melanoma in differential diagnoses of chest wall masses, especially in patients with a recent history of trauma or surgical interventions. The pathophysiology underlying this case may involve a complex interplay between the initial trauma and the subsequent inflammatory processes associated with hemothorax and pyothorax. Trauma-induced local tissue damage could have created a microenvironment conducive to melanoma cell growth, possibly facilitated by inflammatory cytokines and mediators released during the healing process. Additionally, surgical interventions might have disrupted local immune surveillance, allowing melanoma cells to proliferate unchecked.

From a clinical practice perspective, this case underscores the necessity for heightened vigilance and thorough investigation of atypical chest wall swellings. Clinicians should maintain a high index of suspicion for malignancies, including melanoma, even in the absence of classic risk factors or typical presentation [17]. For future research, this case opens avenues for exploring the potential links between trauma, inflammation, and melanoma development. Investigating the molecular and immunological changes in the post-traumatic microenvironment could yield valuable insights into the mechanisms driving melanoma progression and identify novel therapeutic targets.

## Conclusions

This case study illustrates a rare presentation of malignant melanoma as a chest wall swelling in a young male with a history of trauma and subsequent management for hemothorax and pyothorax. The progression from initial traumatic injury to the emergence of a rapidly enlarging, painless chest mass ultimately led to the diagnosis of malignant melanoma, highlighting the aggressive nature of this malignancy. The importance of this case lies in its deviation from typical melanoma presentations. It underscores the necessity for heightened awareness and thorough investigation of atypical chest wall masses, even in patients with no classic risk factors. This case challenges conventional diagnostic expectations and demonstrates that melanoma can manifest in unusual anatomical sites and contexts, particularly following significant trauma and inflammatory conditions. The findings emphasize the need for clinicians to maintain a high index of suspicion for malignancies in patients presenting with unusual symptoms or mass formations. Furthermore, this case suggests a potential link between trauma, inflammatory responses, and melanoma development, warranting further research into the underlying mechanisms. Enhancing diagnostic approaches and broadening differential diagnoses will improve early detection and treatment, ultimately advancing patient care and outcomes in the management of rare and aggressive malignancies.
